# Performance of Three Reflectance Calibration Methods for Airborne Hyperspectral Spectrometer Data

**DOI:** 10.3390/s90200794

**Published:** 2009-02-03

**Authors:** Tomoaki Miura, Alfredo R. Huete

**Affiliations:** 1 Department of Natural Resources and Environmental Management, College of Tropical Agriculture and Human Resources, University of Hawaii at Manoa, 1910 East-West Road, Honolulu, Hawaii 96822, USA; 2 Department of Sol, Water and Environmental Science, University of Arizona, 1177 East Fourth Street, Tucson, Arizona 85721, USA; E-Mail: ahuete@email.arizona.edu

**Keywords:** Reflectance retrieval, hyperspectral reflectance, airborne remote sensing, sensor calibration, radiometry

## Abstract

In this study, the performances and accuracies of three methods for converting airborne hyperspectral spectrometer data to reflectance factors were characterized and compared. The “reflectance mode (RM)” method, which calibrates a spectrometer against a white reference panel prior to mounting on an aircraft, resulted in spectral reflectance retrievals that were biased and distorted. The magnitudes of these bias errors and distortions varied significantly, depending on time of day and length of the flight campaign. The “linear-interpolation (LI)” method, which converts airborne spectrometer data by taking a ratio of linearly-interpolated reference values from the preflight and post-flight reference panel readings, resulted in precise, but inaccurate reflectance retrievals. These reflectance spectra were not distorted, but were subject to bias errors of varying magnitudes dependent on the flight duration length. The “continuous panel (CP)” method uses a multi-band radiometer to obtain continuous measurements over a reference panel throughout the flight campaign, in order to adjust the magnitudes of the linear-interpolated reference values from the preflight and post-flight reference panel readings. Airborne hyperspectral reflectance retrievals obtained using this method were found to be the most accurate and reliable reflectance calibration method. The performances of the CP method in retrieving accurate reflectance factors were consistent throughout time of day and for various flight durations. Based on the dataset analyzed in this study, the uncertainty of the CP method has been estimated to be 0.0025 ± 0.0005 reflectance units for the wavelength regions not affected by atmospheric absorptions. The RM method can produce reasonable results only for a very short-term flight (e.g., < 15 minutes) conducted around a local solar noon. The flight duration should be kept shorter than 30 minutes for the LI method to produce results with reasonable accuracies. An important advantage of the CP method is that the method can be used for long-duration flight campaigns (e.g., 1-2 hours). Although this study focused on reflectance calibration of airborne spectrometer data, the methods evaluated in this study and the results obtained are directly applicable to ground spectrometer measurements.

## 1. Introduction

Several research studies have employed field spectrometers on airplanes to obtain hyperspectral reflectance data for optical characterizations of forest canopies and savanna landscapes [1-3]. These and other studies have found high utility of airborne spectrometers and radiometers because (1) they can rapidly obtain data over rather large areas, encompassing various land cover conditions [2,4], (2) they can measure reflectance factors of tall objects that can not be reached from ground [1], and (3) they can be used to create a reference hyperspectral reflectance database [5].

An issue associated with airborne spectrometers is the conversion of airborne hyperspectral data to reflectance factors, or reflectance calibration. Airborne radiometers or spectrometers are typically flown at low altitudes (<150 m above ground level), “below atmosphere”, to reduce atmospheric effects to a negligible level [2,4]. In calibrating the airborne multispectral data, past studies have used another radiometer on the ground that typically was the same model as, and cross-calibrated to the airborne one. This “ground” radiometer continuously measured radiances reflected off a white reference panel during the flight, to later convert airborne data to reflectance factors by ratioing the two measurement datasets [6-7]. For the airborne hyperspectral spectrometer, however, it is reasonable to presume that having another spectrometer solely dedicated for continuous measurements of incoming solar radiation on the ground, is often too expensive and/or too valuable to afford [5].

In the past, three methods for reflectance calibration of airborne hyperspectral data have been developed and/or used. First, Remer *et al.* [1] used a field spectroradiometer in a “reflectance” mode by calibrating it against a white barium sulfate plate immediately before mounting on the aircraft. In this “reflectance mode” method, incoming solar irradiances were assumed unchanged during the course of the flight campaign. Second, Ferreira *et al.* [2] acquired airborne spectrometer data in an “uncalibrated, raw digital number” mode. The airborne data were calibrated to reflectance factors after the flight by taking a ratio to reference values that were linearly interpolated from the readings made over a Spectralon® white reference panel before and after the flight [2]. In this “linear-interpolation” method, incoming solar irradiances were assumed linearly changed. Finally, Miura *et al.* [5] recently introduced a new reflectance calibration method for airborne spectrometer data. This new method used reference panel readings continuously made with a multi-spectral radiometer at every second on the ground during a flight to “adjust” the panel readings made with a spectrometer before and after the flight and, hence, was referred to as the “continuous panel” method.

Although Miura *et al.* [5] compared the performances of these three reflectance calibration methods, the characteristics of the three methods, in terms of errors and uncertainties in the retrieved reflectance factors, have not been investigated and, thus, are not well known. In this study, we investigated the performances of the three reflectance calibration methods by computing and characterizing errors and uncertainties in the retrieved reflectance factors for various flight scenarios. In the next section, we present our review of the three reflectance calibration methods with their theoretical backgrounds and assumptions used in each of the methods. The datasets and analysis methods used in this study are described in Section 3. Results of the data analyses are presented in Section 4. In the last section (Section 5), we summarize the performance characteristics of the three methods, including their limitations and uncertainty estimates, and conclude with recommended uses of each reflectance calibration method and discussions.

## 2. Background

The reflectance factor is defined as the ratio of the radiant flux reflected by a surface to that reflected into the same reflected-beam geometry by an ideal (lossless), perfectly diffuse (Lambertian) standard surface irradiated under the same conditions [8]. Using a field spectrometer, the reflectance factor for the unknown surface, *R_T_*, is determined from:

(1) $$R_{T}(\theta_{t})=\frac{DN_{T}(t)}{DN_{R}(t)}R_{R}(\theta_{t}),$$

where *θ_t_* is the solar zenith angle at the time *t* and the spectrometer's optical axis is parallel to the surface normal (i.e., nadir-looking geometry); *DN_T_*(*t*) and *DN_R_*(*t*) are the digital numbers from the spectrometer (with the dark current subtracted) when the instrument is viewing the target and reference, respectively, at the time *t; R_R_* is the reflectance factor of the reference panel with respect to a Lambertian surface of unit reflectance [9].

The issue associated with airborne field spectrometer measurements is the difficulty in obtaining *DN_R_* that corresponds to the time when *DN_T_* is recorded. In the following, we review and contrast the three methods evaluated in this study using the same nomenclature as the one used in (1).

### 2.1. Reflectance Mode Method

The reflectance mode method is probably the simplest approach for calibrating airborne measurements to reflectance factors [1]. This method can be expressed as:

(2) $$R_{T}^{RM}(\theta_{t})=\frac{DN_{T}(t)}{DN_{R}(t_{0})}R_{R}(\theta_{t_{0}}),$$

where *t*_0_ is the time immediately before the flight and the superscript *RM* on *R_T_* indicates that the reflectance factor is derived using the reflectance mode method.

In this method, any airborne measurements [i.e., *DN_T_*(*t*)] are divided by *DN_R_*, acquired not at the same time as the airborne measurements, but before the flight [i.e., *DN_R_*(*t*_0_)]. Therefore, the derived reflectance factors, $R_{T}^{RM}$, should be subject to bias errors due to different incoming solar irradiance levels for the reference and airborne measurements. $R_{T}^{RM}$ can be higher or lower than true reflectance factors *R_T_* depending whether the airborne measurements are made before or after the local solar noon and these differences or errors should become larger for longer-time flights. In addition, Miura *et al.* [5] showed that the spectral signatures retrieved by this method were distorted, particularly, over the wavelength regions on which water vapor absorption has a strong impact. Overall, the reflectance mode method is expected to produce reasonable results only for short-term flight campaigns.

### 2.2. Linear-interpolation Method

The linear-interpolation method improves upon the reflectance mode method by estimating *DN_R_* at the time of the target measurement *t* by linearly interpolating *DN_R_* recorded before and after the flight at the time *t*_0_ and *t_e_*, respectively [2]. This method can be expressed as:

(3) $$R_{T}^{LI}(\theta_{t})=\frac{DN_{T}(t)}{DN_{R}^{*}(t)}R_{R}(\theta_{t}),$$

and

(4) $$DN_{R}^{*}(t)=DN_{R}(t_{0})\frac{t_{e}-t}{t_{e}-t_{0}}+DN_{R}(t_{e})\frac{t-t_{0}}{t_{e}-t_{0}},$$

where DN*_R_(*t*) is the linear-interpolated *DN_R_* for the time *t* as in (4) and the superscript *LI* on *R_T_* in (3) indicates that the reflectance factor is derived with the linear-interpolation method.

By predicting *DN_R_*(*t*), this method tries to account for temporal changes in incoming solar irradiances, atmospheric conditions, and optical path lengths through the atmosphere. However, changes in these factors are assumed linear and, thus, any nonlinear changes in them are expected to propagate and result in bias errors in the derived reflectance factors. The magnitudes of these bias errors should change with the time of day and length of the airborne measurement campaign.

### 2.3. Continuous Panel Method

The continuous panel method is the most sophisticated reflectance calibration method among the three and can be considered as an advanced form of the linear-interpolation method. This method computes the “correction factor” to adjust the linearly-interpolated DN*_R_(*t*) for non-linear changes in incoming solar irradiances and optical path lengths through the atmosphere using the continuous panel readings made with a multi-band radiometer [5]:

(5) $$R_{T}^{CP}(\theta_{t})=\frac{DN_{T}(t)}{D{\text{N}}_{R}^{C}(t)}R_{R}(\theta_{t}),$$

and

(6) $$D{\text{N}}_{R}^{C}(t)=DN_{R}^{*}(t)\cdot CF(t),$$

where $R_{T}^{CP}$ is the reflectance factor derived with the continuous panel method and $D{\text{N}}_{R}^{C}(t)$ is the adjusted or corrected $DN_{R}^{*}(t)$ by the correction factor, *CF*(*t*). A brief description of the derivation of this correction factor, including cross-calibration between the multi-band radiometer and spectrometer, is provided in Appendix 1 of this paper. Readers should refer to [5] for the full theoretical description and validation results of the continuous panel method.

Because of the correction factor, the derived spectral reflectance factors are subject mainly to non-linear and/or short-term variations in atmospheric conditions. Therefore, this method is expected to be applicable to long-term flight campaigns. Accuracy of the derived reflectance was estimated at 0.005±0.005 reflectance units for a 3.5 hours flight scenario [5].

## 3. Materials and Methods

Two datasets were used to characterize and compare error, accuracy, and precision of the three reflectance calibration methods. One dataset was acquired in a ground-based field experiment that enabled us to simulate airborne measurement protocols for various flight scenarios. An airborne campaign was conducted to obtain the other dataset, in which a spectrometer was actually flown to measure reflected radiation from a tropical landscape.

### 3.1. Field Experiment

The field experiment was conducted in the Jornada Experimental Range in the United States on October 5, 2002. The sky was clear and cloud-free during the experiment. The range is located 37 km north of Las Cruces, New Mexico, in the northern part of the Chihuahuan Desert. The climate is semi-arid, with a mean annual precipitation of 210 mm and a mean annual temperature of 16 °C. The range was once black grama (*Bouteloua eriopoda*)-dominated grasslands. Due to heavy grazing and fire suppression, however, the grasslands have been transformed into dune-like mesquite (*Prosopis glandulosa*) shrublands and creosote bush (*Larrea tridentate*) communities [10-12].

A 100 m transect was drawn in the north-south direction on one of the remaining grassland patches within the range (N 32.58914°/W 106.84277°, 1,330 m elevation). Most of plants were black grama, but there were also several yucca (*Yucca elata*) plants. Near the south end of the transect, a four-band radiometer (Exotech Model 100BX) was setup on a tripod to nadir-look over a leveled Spectralon^®^ white standard panel surface, 30 cm below (Labsphere, Inc., North Sutton, NH, USA). With a 15 degree field-of-view (FOV) lens attached, an 8 cm-by-8 cm area of the panel was viewed by each of the four Exotech bands. The bandpasses of this Exotech radiometer were designed to approximate the first four bands of Moderate Resolution Imaging Spectroradiometer (MODIS); Band 1, 456 – 475 nm; Band 2, 544 – 564 nm; Band 3, 623 – 670 nm; and Band 4, 838 – 876 nm [13]. Panel-reflected radiant energy sensed by the radiometer as analog voltage signals were recorded as four decimal precision numbers every 15 seconds from 9:20 a.m. to 1:20 p.m., MST, bracketing local solar noon of the day (11:55 a.m. MST). The signal-to-noise ratio (SNR) of the Exotech continuous panel data ranged from 500 to 900, depending on the time of day.

A full-range hyperspectral field spectrometer [Analytical Spectral Devices (ASD), Inc., Boulder, CO, USA] was used to measure reflected radiation along the transect. The ASD spectrometer acquires spectral data in 1.4 nm intervals in the visible/NIR [380 – 1,300 nm; full-width at half-maximum (FWHM) = 3 – 4 nm] and 2.2 nm in the SWIR region (1300 – 2450 nm; FWHM = 10 – 12 nm) that are recorded as 16-bit digital numbers by an attached computer. The ASD spectrometer contains three subspectrometers inside, each of which operates on different wavelength regions: the first VNIR subspectrometer measures light between 380 – 972 nm, the second SWIR1 subspectrometer covers the region between 972 – 1,767 nm, and the last SWIR2 subspectrometer covers the remaining region. “Optimization” is periodically performed on the instrument to adjust sensitivity of the three subspectrometers to varying conditions of illumination. Whereas dark current (DC) measurements are performed at every scan for the SWIR1 and SWIR2 subspectrometers, they are performed only at optimizations or upon a user's request for the VNIR subspectrometer. This difference in the DC correction schemes between the VNIR and the other two subspectrometers often results in a glitch or discontinuity at 972 nm in the acquired spectra. The SNR of the ASD spectrometer varies across wavelengths and can also change depending on the instrument/spectral data collection setting used. For the setting used in this study, SNR were ∼1,500 for the VNIR region, ∼1,000 for the SWIR1 region, and 200 – 500 for the SWIR2 region.

An 18 degree foreoptic was attached to the “fiberoptic gun” extended from the spectrometer which was mounted on a backpack “yoke” and held at 1.5 m above ground. This produced an approximately 47 cm-by-47 cm ground spatial resolution. Transect measurements were made by moving first from the south end to the north end of the transect and then from the north to south ends. On the transect, ten scans were made and averaged to record one spectrum every 2 m, resulting in 100 spectra recorded per transect run. Before and after the transect measurements, reference data were collected over another leveled Spectralon® white standard panel and placed near the Exotech radiometer. This sequence of measurements (i.e., panel, transect, and panel) took 10 minutes to complete and was repeated for eight times every 30 minutes, starting at 9:30 a.m.

### 3.2. Airborne Measurements

The airborne campaign was conducted in a tropical forest-savanna transitional zone near Santana do Araguaia in Brazil (S 10°/W 50°) on July 26, 2001 (dry season). This study area represented a variety of land cover types, including undisturbed forest and savanna vegetation formations. The mean annual precipitation and mean annual temperature in this area are 1,670 mm and 26 °C, respectively.

An ASD field spectrometer equipped with a GPS device (GPS III+, Garmin International, Inc., Olathe, KS, USA) and a digital camera (C3000, Olympus Imaging America, Inc., Center Valley, PA, USA) were flown “below the atmosphere” at 150 m AGL at the speed of 30 m per second. A 5 degree foreoptic was attached to the spectrometer, and 10 spectra were scanned and internally averaged for a single spectrum collection at every ∼1 second. This resulted in the ground spatial resolution of 13 m-by-30 m. ASD spectrometer readings over a Spectralon® reference panel were made before and after the flight. An Exotech 100BX radiometer was setup in the middle of an open field for the continuous panel data collection. The flight and ground crews constantly communicated so that the continuous panel data period completely bracketed that of the airborne data including the preflight and post-flight ASD panel readings.

The flight duration was approximately 2 hours where the actual target measurements were made across forest and savanna landscapes from 9:40 a.m. (GMT – 3 hours) for 1 hour and 20 minutes. The preflight and postflight ASD panel readings were made at 9:15 a.m. and 11:30 a.m., respectively (approximately 30 minutes before and after the airborne measurement period).

### 3.3. Data Analysis Methods

We first investigated the performance characteristics of the three calibration methods for one hour flight scenarios conducted at different times of day using the field experimental dataset. Data from six transect runs (10:00-10:10 a.m., 10:30-10:40 a.m., 11:00-11:10 a.m., 11:30-11:40 a.m., 12:00-12:10 p.m., and 12:30-12:40 p.m., MST) were processed into reflectance factors by the three reflectance calibration methods. In order to simulate these one-hour flight scenarios, data from each transect run was processed using ASD plate readings collected 30 minutes before and/or after that transect run and, thus, the derived spectra were considered airborne measurements obtained in the middle of one hour flights. The continuous panel data obtained with the Exotech radiometer were also used in the continuous panel method. “True” reflectance factors for each transect run were obtained using the ASD panel measurements made immediately before and after the corresponding transect run with the linear-interpolation method (a standard protocol for ground reflectance measurements).

Three statistical measures of accuracy and precision were used to characterize the performances of the three reflectance calibration methods. First, mean differences (MD) were computed to examine bias errors in the reflectance factors derived using each of the three methods:

(7) $$\begin{array}{ll} MD & =\frac{1}{n}\sum_{i=1}^{n}\left(R_{T,\;i}^{*}-R_{T}\right)\\ & =\frac{1}{n}\sum_{i=1}^{n}e_{i}, \end{array}$$

where $R_{T}^{*}$ is the reflectance factor derived by one of the reflectance calibration methods, *n* is the sample size, and *e* is the calibration error defined as the retrieved reflectance value minus the true reflectance value ( $R_{T}^{*}-R_{T}$). Second, root mean square errors (RMSE) were used as an indicator of accuracy of the reflectance calibration methods:

(8) $$\begin{array}{ll} \text{RMSE} & =\sqrt{\frac{1}{n}\sum_{i=1}^{n}{\left(R_{T,\;i}^{*}-R_{T}\right)}^{2}}\\ & =\sqrt{\frac{1}{n}\sum_{i=1}^{n}{\left(e_{i}\right)}^{2}}. \end{array}$$

Finally, we computed the standard deviations of the calibration error, *e*, (STD) as a quantitative measure of the variability of *e* about MD, or the precision in the reflectance calibration results:

(9) $$\text{STD}=\sqrt{\frac{1}{n}\sum_{i=1}^{n}{\left(e_{i}-MD\right)}^{2}}.$$

Second, we investigated the performances of the calibration methods for various flight lengths. We processed the field transect data acquired at 11:00-11:10 a.m. MST, but using ASD plate readings made at 20, 30, 50, 60, 80, and 90 minutes before and/or after the transect measurement. These simulated 50 minutes, 1 hour 10 minutes, 1 hour 50 minutes, 2 hours 10 minutes, 2 hours 50 minutes, and 3 hours 10 minutes flight scenarios, respectively. The same statistical measures of accuracy and precision were computed for all of the 18 reflectance datasets (the three reflectance calibration methods applied for the six flight scenarios) and compared across the methods and flight hours. The true reflectance factor values for the 11:00-11:10 a.m. MST transect run derived in the first analysis were used as the true, reference reflectance factor values for this analysis.

Finally, we verified some of the characteristics of the reflectance calibration methods determined in the above two analyses using the airborne dataset. The airborne spectral data were first converted to reflectance factors with each of the three calibration methods. We then extracted reflectance spectra acquired at various points of time during the flight, but over the same land cover types of forest and savanna. High-resolution digital camera images collected along with the spectral data were used to assure that the extracted spectra were obtained for the same land cover types. Spectral shapes and absolute reflectance values of these extracted reflectance spectra were compared across the methods and acquisition times.

## 4. Results

### 4.1. Performance Characteristics across Different Times of Day

**Figure 1.**
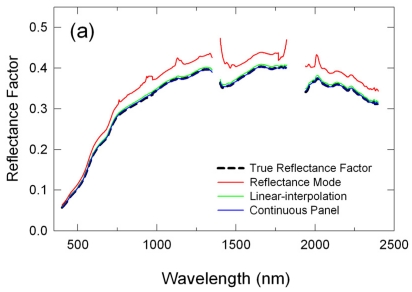

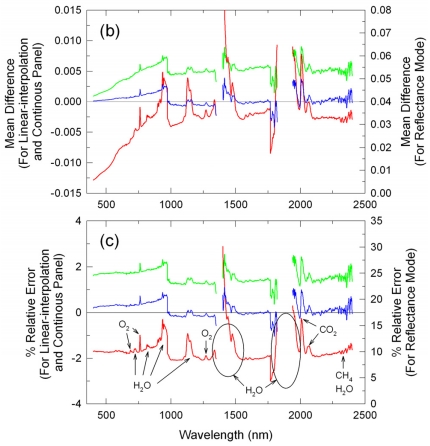
Mean reflectance spectra for the 10:30 a.m. MST transect run derived with three reflectance calibration methods: (a) reflectance factors, (b) mean differences, and (c) % relative mean differences. For (b) and (c), the y-axes on the left are for the linear-interpolation and continuous panel results and the y-axes on the right are for the reflectance mode method results.

In Figure 1, mean reflectance spectra obtained by the three reflectance calibration methods are plotted as an example of the derived reflectance spectra. These are for the 10:30 a.m. transect run for the one hour flight scenario and, thus, the ASD panel readings made at 10:00 a.m. MST were used in the reflectance mode (RM) method, whereas those not only at 10:00 a.m. but also at 11:10 a.m. MST were used in both the linear-interpolation (LI) and continuous panel (CP) methods.

All three methods retrieved a convex spectral signature that was overall similar to that of the true reflectance spectrum with the lowest and highest reflectance factors in the blue and SWIR regions, respectively (Figure 1a). However, the retrieved reflectance factors by the RM and LI methods were higher than the true reflectance factors. On average, the RM-derived reflectances were higher by 0.03 reflectance units (10%) and the LI-derived reflectances were higher by 0.005 reflectance units (2%) than the true reflectances (Figures 1b,c). On the other hand, the CP-derived reflectances were unbiased, being basically equal to the true reflectances (Figures 1b,c).

The derived reflectance spectra by the three methods were all subject to bias errors due to absorptions by several atmospheric constituents, including water vapor (H_2_O), oxygen (O_2_), carbon dioxide (CO_2_), and methane (CH_4_) (Figure 1c). Their impacts were the largest on the RM method since it did not account for any changes in atmospheric conditions and atmospheric path lengths. It should be noted that one can see glitches at around 970 nm in the mean difference (MD) spectra (Figures 1b,c), which were due to drifts in the ASD spectrometer as described in Section 3.1.

**Figure 2.**
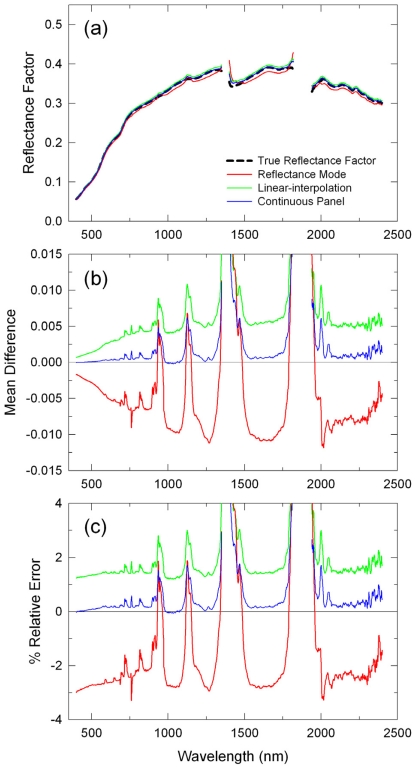
Same as Figure 1, but for the 12:30 p.m. MST transect run.

In Figure 2, another set of the derived mean reflectance spectra for the 12:30 p.m. transect run, acquired after local solar noon, are plotted for comparisons to those in Figure 1. Several differences in the calibration results were observed between these two. The RM-derived reflectances were smaller than the true reflectance factors (negative errors: at -0.01 reflectance units or 3% relative level) although these errors were smaller than those for the 10:30am transect run (Figures 2b,c). On the other hand, errors in the LI- and CP-derived reflectances were about the same as for the 10:30 a.m. transect run: LI results on average 0.005 (2%) and CP results nearly zero (Figures 2b,c). The impact of atmospheric absorptions appeared to be larger on this transect run due most likely to a larger change in atmospheric conditions (gaseous absorptions).

**Figure 3.**
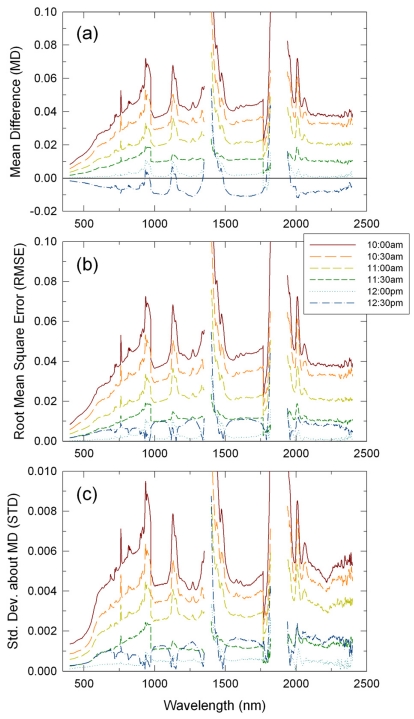
Changes in performance statistics of the reflectance mode method across different time of day: (a) mean differences (MD), (b) root mean square errors (RMSE), and (c) standard deviations about MD (STD). These are for one hour flight scenarios.

**Figure 4.**
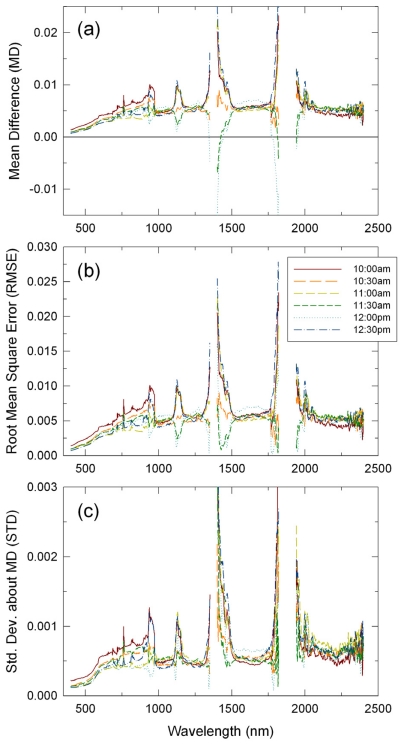
Same as Figure 3, but for the linear-interpolation method.

**Figure 5.**
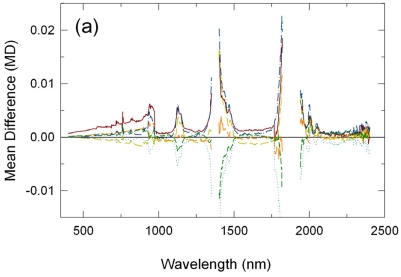

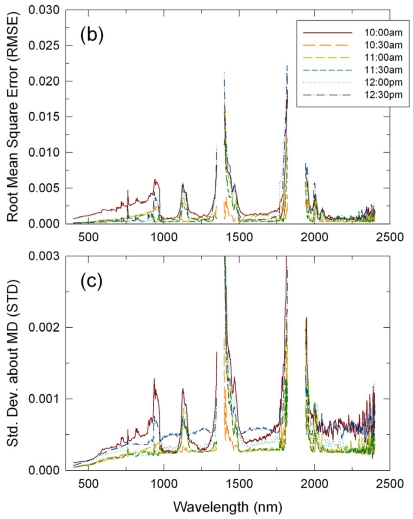
Same as Figure 3, but for the continuous panel method.

In order to more thoroughly characterize changes in the performances of those methods within a day, MD, RMSE, and STD spectra for all the six transect runs (six different times of day) are plotted for each of the RM, LI, and CP methods in Figures 3, 4 and 5, respectively. MD of the RM-derived reflectance spectra changed significantly, but systematically across the six transect runs (Figure 3a). MD were the largest at 0.04 reflectance units on average for the 10:00 a.m. run, became smaller for the other morning transect runs, were nearly zero for the 12:00 p.m. run, and then became negative at -0.01 reflectance units on average for the 12:30 p.m. run. The other two statistics of RMSE and STD showed the same trend, except that they were also positive for the 12:30 p.m. run because both RMSE and STD are definite positive (Figures 3b,c). Gaseous absorptions affected earlier morning datasets more significantly (Figures 3a,b,c).

For the LI-derived reflectance factors, MD were at the same level of 0.005 reflectance units, on average, for all the six transect runs (Figure 4a). For the 10:00 a.m. transect run, MD, RMSE, and STD were slightly larger in the 400 – 950 nm wavelength range than for the other runs (Figures 4a,b,c). This was attributed to drifts in the ASD VNIR subspectrometer. There was no systematic trend in the effects of gaseous absorptions on the LI results and their effects appeared as either convex or concave residual absorption features in the MD, RMSE, and STD spectra (Figures 4a,b,c).

The CP methods resulted in the smallest MD, RMSE, and STD (Figures 5a,b,c). In particular, MD were on average zero and, although there was some variation in MD across the transect runs, there was no systematic trend in the MD variation (Figure 5a). MD, RMSE, and STD for the 10:00 a.m. transect run were slightly larger than those for the other runs. This was due to errors in empirical cross-calibration between the spectrometer and the radiometer for this particular transect run for the derivation of correction factor values or due to drifts in the ASD VNIR subspectrometer (Eq. 6; see also Appendix 1). For all the three reflectance calibration methods, RMSE were basically of the same magnitudes as MD (Figures 3b, 4b, and 5b), whereas STD were approximately 10 % of MD (Figures 3c, 4c, and 5c).

In summary, the RM-derived reflectance factors were generally biased and subject to larger bias errors when the flight campaign was conducted either earlier in the morning or later in the afternoon, even for the one hour flights. The RM method could produce unbiased results only when the flight campaign occurred around local solar noon. The LI-derived reflectance factors were subject to the same level of bias errors regardless of time of day. The CP method was the only reflectance calibration method that retrieved unbiased reflectance factors for any time during a day.

### 4.2. Performance Characteristics across Different Flight Time Lengths

**Figure 6.**
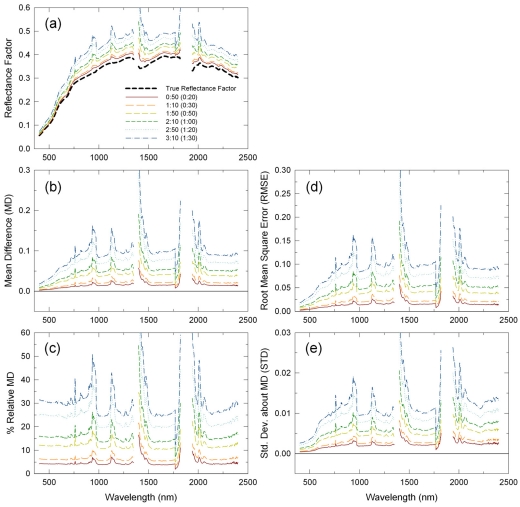
Changes in performance statistics of the reflectance mode method across different flight durations (time lengths): (a) mean reflectance factors, (b) mean differences (MD), (c) % relative MD, (b) root mean square errors (RMSE), and (c) standard deviations about MD (STD). The numbers in parentheses in the legends are the time in minutes between the preflight panel readings and the target measurements.

**Figure 7.**
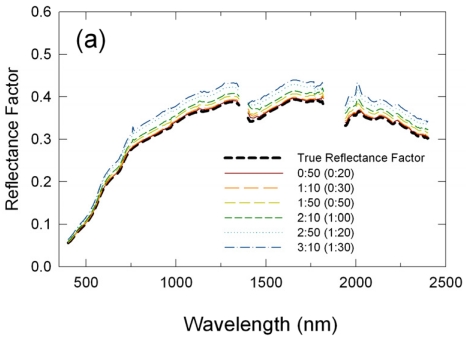

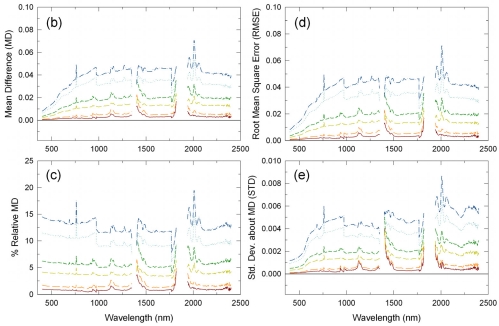
Same as Figure 6, but for the linear-interpolation method.

**Figure 8.**
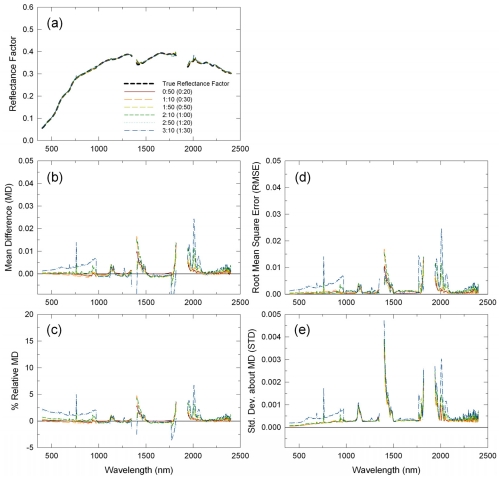
Same as Figure 6, but for the continuous panel method.

Retrieved reflectance spectra and their statistical measures of accuracy and precision for all the six simulated flight time lengths are plotted for the RM, LI, and CP methods in Figures 6, 7, and 8, respectively. The RM-retrieved reflectance spectra were biased with errors increasing with an increase in the flight time length (Figure 6). MD were positive for all the simulated flight lengths, indicating that the RM-retrieved reflectances were always higher than the true values (Figure 6a). MD were, on average, 0.02 reflectance units (5 % relative MD) for the shortest simulated flight length of 50 minutes, but increased to 0.1 reflectance units (25-30 % relative MD) for the longest simulated flight length of 3 hours 10 minutes (Figures 6b,c). RMSE showed the same behavior as MD (Figure 6d). STD also increased with an increase in the flight length, indicating that variations in reflectance retrieval errors were larger for longer flights (Figure 6e). STD were about 10% of MD or RMSE. Distortions in the derived spectral signatures due to atmospheric absorptions also increased with increasing flight durations (Figure 6). This was primarily because differences in the atmospheric path lengths between the time of the calibration and airborne measurements became larger for longer flight durations, with temporal variability in atmospheric conditions imparting a secondary contribution to the distortions.

The behavior patterns of the LI-retrieved reflectance errors were very similar to those of the RM-retrieved reflectance errors, but with smaller magnitudes (Figure 7). MD were less than 0.005 reflectance units (< 2 % relative MD) for the shortest simulated flight length and increased to more than 0.04 reflectance units (> 12 % relative MD) for the longest simulated flight length (Figure 7a, b), which were less than half a magnitude of the RM-retrieval errors. RMSE were about the same level as MD (Figure 7d). STD were about 10 % of MD and RMSE, and increased with an increase in the flight length (Figure 7e). The LI-retrieved spectral signatures were subject to much lower distortions due to atmospheric gaseous absorption, indicating that linear-interpolations of the two plate readings successfully reduced atmospheric sources of errors due to path length differences (Figure 7). On the LI-retrieved reflectance spectra, atmospheric constituents had variable effects. Absorption effects due to oxygen (O_2_) and carbon dioxide (CO_2_) increased with increasing flight lengths, whereas the impacts of water vapor (H_2_O) absorptions did not necessarily increase with an increase in flight length (Figure 7). These were associated with temporal variability of atmospheric conditions.

The CP method consistently derived accurate, unbiased reflectance factors regardless of flight lengths (Figure 8). MD and RMSE were basically zero for all the simulated flight lengths, except for the VNIR reflectances that were biased for several simulated flight lengths due to drifts in the ASD instrument (Figures 8a,b,c,d). STD were, on average, 0.0025 reflectance units for all the six simulated flight lengths, indicating that, unlike the other two methods, variability in CP retrieval results was the same regardless of flight length (Figure 8e). The impacts of atmospheric absorptions on the CP-retrieved reflectances were the same as those on the LI-retrieved reflectances.

### 4.3. Performance Evaluation with Tropical Forest and Savanna Dataset

**Figure 9.**
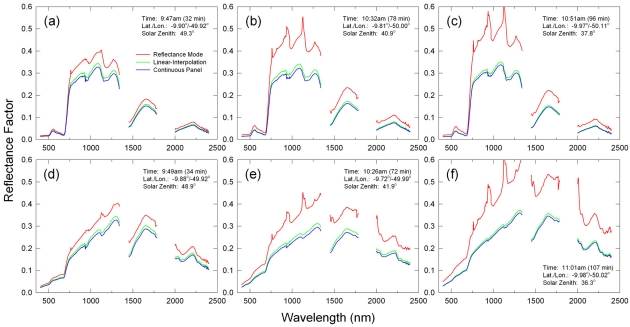
Comparison of reflectance spectra derived with three different reflectance calibration methods obtained at the beginning of the airborne measurements (∼30 minutes after the preflight ASD reference panel readings) for (a) tropical forest and (d) savanna, at the middle of the flight (∼75 minutes after the preflight panel readings) for (b) tropical forest and (e) savanna, and at the end of the flight (∼100 minutes after the panel readings) for (c) tropical forest and (f) savanna.

Three sets of the extracted spectral data for tropical forests and savannas in Brazil are shown in Figure 9. Although we did not have independent, ground-measured reflectance data to validate the absolute accuracies of these retrieval results from this airborne dataset, the above inter-comparisons of the derived reflectance spectra among the three reflectance calibration methods showed the expected error and uncertainty characteristics from the analysis results obtained in Sections 4.1 and 4.2. Basically, the RM method was not useful at all for converting this airborne dataset into reflectance factors. Toward the end of the flight, RM-derived reflectance factors became higher, while their spectral signatures were subject to more distortions due to atmospheric absorptions, for both forest and savanna land cover types (Figure 9). The LI and CP methods resulted in spectral reflectance retrievals that were much more reasonable, i.e., the only recognizable distortions in the retrieved spectral signatures occurred at the 940 nm and 1,140 nm water vapor absorption regions (Figure 9). These water vapor absorption effects appeared the most significant in the savanna spectra collected at 9:49 a.m. LST (Figure 9d). The LI-derived reflectance factors were always higher than the CP counterparts, of which magnitudes were the highest for the spectral data acquired in the middle of the flight (Figures 9b,e).

## 5. Discussion and Conclusions

In this study, we investigated and compared the performance characteristics of three methods for converting airborne hyperspectral spectrometer data into reflectance factors: the reflectance mode (RM), linear-interpolation (LI), and continuous panel (CP) methods. The RM method was found to be the least useful. By having an airborne spectrometer pre-calibrated before the flight, spectral reflectance data obtained with this method were biased and distorted due to constantly changing incoming solar irradiance levels and atmospheric conditions, respectively. Likewise, the magnitudes of these bias errors and distortions varied significantly, depending on time of day and length of the flight campaign. The RM method can produce reasonable results only for very short-term flights (e.g., < 15 minutes) conducted around local solar noon.

The LI method was found to be a precise, but inaccurate reflectance retrieval method. By using pre-flight and post-flight reference panel readings, the LI method can derive “clean” spectral signatures not distorted by atmospheric absorptions. However, the derived spectral reflectance factors are subject to bias errors with magnitudes dependent not on the time of day in which the flight campaign occurs, but on the flight time length. The flight duration should be kept shorter than 30 minutes for the LI method to produce results with reasonable accuracies.

The CP method was found to be an accurate and reliable reflectance calibration method. The use of continuous panel readings to adjust the magnitudes of linearly-interpolated reference panel readings produced accurate reflectance factors. Likewise, the performances of the CP method in retrieving accurate reflectance factors were consistent throughout time of day and for various flight durations. An important advantage of the CP method is that the method can be used for long-duration flight campaigns (e.g., 1-2 hours). Based on the dataset analyzed in this study, uncertainty of the CP method has been estimated to be 0.0025 ± 0.0005 reflectance units for the wavelength regions not affected by atmospheric absorptions.

In this study, we used a four band Exotech Model 100BX radiometer to obtain continuous panel data. Although the four Exotech bands only covered the visible and NIR wavelength regions (approximately 450 – 880 nm), the derived correction factor values successfully adjusted $DN_{R}^{*}(t)$ in other wavelength regions. This result appears to indicate that changes in incoming solar irradiance levels can be treated as wavelength-independent and that correction factors may be computed with fewer spectral bands as long as continuous panel readings obtained with those bands capture changes in incoming solar irradiance levels well. As seen in Equations A3-A5, the correction factor also accounts for FOV or geometry differences between the spectrometer and the radiometer used. Thus, it may be possible to use a continuous data record obtained with a hemispherical pyranometer to derive correction factors. In this study, we felt that it was advantageous to use a multi-band radiometer because the multi-bands allowed us to evaluate the wavelength-independence of changes in incoming solar irradiance levels and uncertainties in the derived correction factors.

Although this study focused on airborne spectrometer data, the methods used and the results obtained here are directly applicable to ground reflectance measurements with a spectrometer.

## Appendix 1. Derivation of Correction Factor [5]

The derivation of the correction factor for the continuous panel method assumes that (1) there is a field spectrometer to be flown on an airplane and there is a multi-band radiometer for the continuous panel measurements on a ground and (2) there are two calibrated plates: one to be taken to an airport with the field spectrometer and the other to be used on the ground for the continuous measurements.

Because of the uniformity in the reflective properties of the panel surfaces, the readings made with the spectrometer and multi-band radiometer over the panels can be expressed in the irradiance terms (W/m^2^/μm):

(A1) $$DN_{R}(t)=\frac{E_{0}(t)R_{R}(\theta_{i})}{c_{DN}\pi }\int_{\omega_{r}}d\omega_{r},$$

(A2) $$V_{R2}(t)=\frac{E_{0}(t)R_{R2}(\theta_{i})}{c_{V}\pi }\int_{\omega_{r2}}d\omega_{r2},$$

where *V_R_*_2_(*t*) is the DC subtracted voltages from the multi-band radiometer over the continuous panel *R*2 at the time *t; R_R_* and *R_R_*_2_ are the reflectance factors of the two panels; *c_DN_* and *c_V_* are the calibration coefficients for the spectrometer and radiometer, respectively (W/m^2^/μm/DN or W/m^2^/μm/Voltage); *E_0_*(*t*) is the band averaged incoming solar irradiance (W/m^2^/μm) at ground level at the time *t; ω_r_* and *ω_r_*_2_ are the field of view (FOV) of the spectrometer and radiometer, respectively. By Equations A1 and A2, the correction factor derivation further assumes that (3) the difference in the band averaged solar irradiances measured by these two sensors is negligible (i.e., *E*_0_) and (4) the solar zenith angle difference between the airport and the study site have a negligible impact, which is considered reasonable when they are in proximity.

Solving Equation A2 for *E*_0_ and substituting *E*_0_ in Equation A1 derives the following equation:

(A3) $$DN_{R}(t)=\frac{\int_{\omega_{r}}d\omega_{r}}{\int_{\omega_{r2}}d\omega_{r2}}\cdot \frac{c_{V}}{c_{DN}}\cdot \frac{R_{R}(\theta_{i})}{R_{R2}(\theta_{i})}\cdot V_{R2}(t)+\varepsilon ,$$

or

(A4) $$DN_{R}(t)=C_{V\to DN}\cdot \frac{R_{R}(\theta_{i})}{R_{R2}(\theta_{i})}\cdot V_{R2}(t)+\varepsilon ,$$

and

(A5) $$C_{V\to DN}=\frac{\int_{\omega_{r}}d\omega_{r}}{\int_{\omega_{r2}}d\omega_{r2}}\cdot \frac{c_{V}}{c_{DN}},$$

where the error term, *ε*, includes errors that arise from the last two assumptions used in deriving Equation A3 [(3) and (4) above]. This newly-derived coefficient, *C_V_*_→_*_DN_*, is the cross-calibration coefficient that relates *V_R_*_2_ to *DN_R_*, which shows that the overall difference in the two sensors' outputs is due to FOV's and absolute calibration. In practice, this cross-calibration coefficient can be obtained from the coincident measurements made with the two sensors before and after the flight.

Considering that the radiometer has multiple bands, the equations are now expanded to explicitly include the factor. Equation A4 can be used to predict *DN_R_* for the band *b* from *V_R_*_2_:

(A6) $${\overset{\wedge }{DN}}_{R}(b,t)=C_{V\to DN}(b)\cdot \frac{R_{R}(b,\theta_{i})}{R_{R2}(b,\theta_{i})}\cdot V_{R2}(b,t).$$

The predicted *DN_R_* at several discrete spectral bands can be made into a single correction factor value, *CF*(*t*), by assuming that the magnitude changes in the incoming solar irradiance are independent of wavelength:

(A7) $$CF(t)=\frac{1}{n}\sum_{b=1}^{n}\frac{{\overset{\wedge }{DN}}_{R}(b,t)}{DN_{R}^{*}(b,t)},$$

where *DN***_R_* is the linear-interpolated *DN_R_* based on those made before and after the flight at the time *t*_0_ and *t_e_*, respectively (Equation 4).
